# Did the introduction and increased prescribing of antidepressants lead to changes in long-term trends of suicide rates?

**DOI:** 10.1093/eurpub/ckaa204

**Published:** 2020-11-25

**Authors:** Simone Amendola, Martin Plöderl, Michael P Hengartner

**Affiliations:** 1 Department of Dynamic and Clinical Psychology, Faculty of Medicine and Psychology, Sapienza University of Rome, Rome, Italy; 2 Department of Crisis Intervention and Suicide Prevention, Christian Doppler Clinic, Paracelsus Medical University Salzburg, Salzburg, Austria; 3 Department of Applied Psychology, Zurich University of Applied Sciences, Zurich, Switzerland; 4 Medical Faculty, University of Zurich, Zurich, Switzerland

## Abstract

**Background:**

Ecological studies have explored associations between suicide rates and antidepressant prescriptions in the population, but most of them are limited as they analyzed short-term correlations that may be spurious. The aim of this long-term study was to examine whether trends in suicide rates changed in three European countries when the first antidepressants were introduced in 1960 and when prescription rates increased steeply after 1990 with the introduction of the serotonin reuptake inhibitors (SSRIs).

**Methods:**

Data were extracted from the WHO Mortality Database. Suicide rates were calculated for people aged 10–89 years from 1951–2015 for Italy, 1955–2016 for Austria and 1951–2013 for Switzerland. Trends in suicide rates stratified by gender were analyzed using joinpoint regression models.

**Results:**

There was a general pattern of long-term trends that was broadly consistent across all three countries. Suicide rates were stable or decreasing during the 1950s and 1960s, they rose during the 1970s, peaked in the early 1980s and thereafter they declined. There were a few notable exceptions to these general trends. In Italian men, suicide rates increased until 1997, then fell sharply until 2006 and increased again from 2006 to 2015. In women from all three countries, there was an extended period during the 2000s when suicide rates were stable. No trend changes occurred around 1960 or 1990.

**Conclusions:**

The introduction of antidepressants around 1960 and the sharp increase in prescriptions after 1990 with the introduction of the SSRIs did not coincide with trend changes in suicide rates in Italy, Austria or Switzerland.

## Introduction

There is ongoing debate about the impact of antidepressant use on the risk of suicide, at both the individual level and the population level.^1^^,^^2^ Meta-analyses of clinical trials have found either no definite association between antidepressant use and risk of (attempted) suicide or significantly increased risk.^3–7^ Observational studies likewise produced inconsistent results. An older meta-analysis of case–control and cohort studies found increased suicide risk with selective serotonin reuptake inhibitors (SSRIs) in paediatric patients with depression and significantly reduced risk in adult patients.^8^ An updated and expanded meta-analysis found no definite association between SSRIs and suicide risk in adult patients with depression but significantly increased risk with any new-generation antidepressant in adult patients with depression and other treatment indications.^9^

Nevertheless, based on the findings from ecological studies, various authors have concluded that the increased prescription of SSRIs and other new-generation antidepressants has likely contributed to lower suicide rates in the population.^2^^,^^10^^,^^11^ Such strong claims are not firmly supported by the evidence though, as the results of ecological studies are inconsistent. Some studies have found negative correlations between antidepressant prescriptions and suicide rates, some no association, and yet others even positive correlations.^12–14^ However, on the population level, a disease can be highly correlated with an environmental variable even when on an individual level they are unrelated or show an association in the opposite direction, a methodological bias long known as the ecological fallacy.^15^ This bias occurs when the background rate of a disease varies across groups, e.g. different socioeconomic strata.

In one of the most rigorously conducted ecological studies, Dahlberg and Lundin^16^ showed a negative and statistically highly significant correlation between antidepressant prescriptions and the suicide rate between 1990 and 2000 in Sweden when other variables where not controlled for. By contrast, when they controlled for socioeconomic factors, the correlation between suicides and antidepressants was no longer negative and statistically non-significant.^16^

Complicating matters further, most ecological studies have correlated antidepressant prescriptions and suicide rates over a relatively short time-period, commonly between around 1990 and the early 2000s,^11^^,^^12^ even though antidepressants have been used in clinical practice since about 1960. Suicide rates can fluctuate widely over decades due to various socioeconomic and cultural factors.^17–19^ Therefore, restricting the analysis to a short time-period, e.g. 1990–2000, may easily produce spurious correlations, even when known confounders, such as unemployment and alcohol consumption, are statistically controlled for.^20^

The aim of this study was to analyze long-term trends in suicide rates in Italy, Switzerland and Austria. These three countries were chosen as they are the countries of origin of the study authors and appropriate long-term suicide data were available. Moreover, the three countries have similar healthcare systems (statutory health insurance; general practitioners act as gatekeepers), but otherwise differ culturally (Italy is a Mediterranean Latin country, Austria an Alpine Germanic country and Switzerland a mixed Latin-Germanic country). To avoid the methodological biases detailed above, we did not compute correlations between suicide rates and antidepressant prescriptions, but examined whether the introduction of the first antidepressants in clinical practice around 1960^21^ and the steep increase in prescriptions after 1990 with the introduction of the SSRIs and other new-generation antidepressants^11^ would relate to sustained changes in long-term trends in suicide rates. As outlined by Stone,^22^ reductions in suicide rates should mostly occur when new antidepressants are first introduced into a population, but there will be much less reduction as use spreads. As a result, assuming antidepressant prescriptions had a clear and sustained suicide-protective effect at the population level, we would expect (i) a decrease in suicide rates around 1960 when the first antidepressants were introduced and (ii) another decrease starting 1990 when the SSRIs were introduced.

## Methods

### Data sources

Suicide deaths among people aged 10–89 years in Italy, Austria and Switzerland were examined. The annual data on suicides were extracted in March 2020 from the WHO Mortality Database (https://apps.who.int/healthinfo/statistics/mortality/causeofdeath_query/start.php) for the periods 1951–2015 for Italy (64 years), 1955–2016 for Austria (61 years) and 1951–2013 for Switzerland (62 years), overall and stratified by gender. Suicide as a cause of death was recorded according to the seventh (code A148, suicide and self-inflicted injury), eighth (code A147, suicide and self-inflicted injury), ninth (codes E950–E959, suicide and self-inflicted injury) and tenth (codes X60–X84, intentional self-harm) revision of the International Classification of Diseases. Based on the literature, we set the time point for the introduction of the first antidepressants in clinical practice around 1960^21^^,^^23^ and the introduction of the SSRIs that prompted a steep and mostly ongoing increase in antidepressant prescriptions in Europe and elsewhere as of 1990.^11^^,^^24^^,^^25^

According to Swiss law, the study was exempt of approval by an ethics committee because all data were de-identified and publicly available.

### Statistical analysis

Suicide rates were calculated as the annual number of suicides per 100 000 individuals (age range 10–89 years), overall and by gender. Trends in suicide rates were analyzed using the Joinpoint Regression Program, version 4.8.0.1, from the US National Cancer Institute.^26^ Joinpoints were determined separately by gender. These models were used to estimate the average annual percent change (APC) in suicide rates and the number and location of joinpoints, based on linear regression with the log suicide rates as the dependent variable and the year as the independent variable. The regression model assumed that the random errors followed a Poisson distribution and the regression coefficients were estimated by weighted least squares.^26^ Joinpoint regression performs multiple tests in order to select the number of joinpoints using permutation test and choosing the model with the best fit to the data.^27^ In the final model, joinpoints indicate the points in time when changes in long-term trends occur. The APC, based on the slope of the line segment, describes an increasing trend (positive APC value) or decreasing trend (negative APC value).

## Results

### Suicide rates

**Figure 1:**
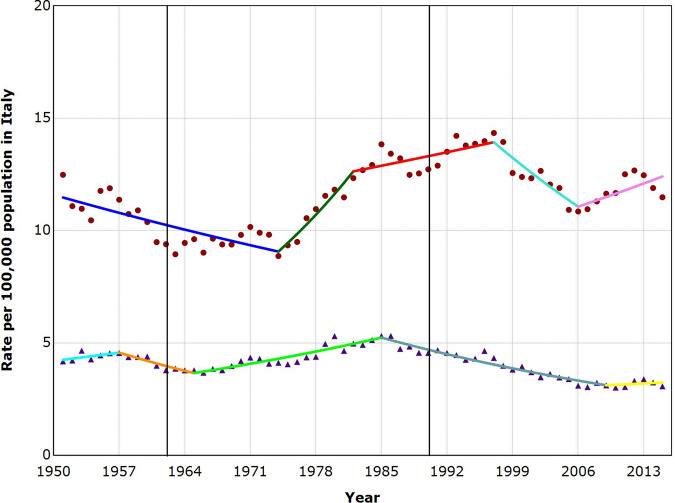
Suicide trends among males and females aged 10–89 years in Italy, 1951–2015 Dots, male suicide rate; Triangles, female suicide rate. The two bold vertical lines denote the time points when the first antidepressants were introduced (1960) and when the SSRIs were introduced and widespread prescription began (1990).

**Figure 2:**
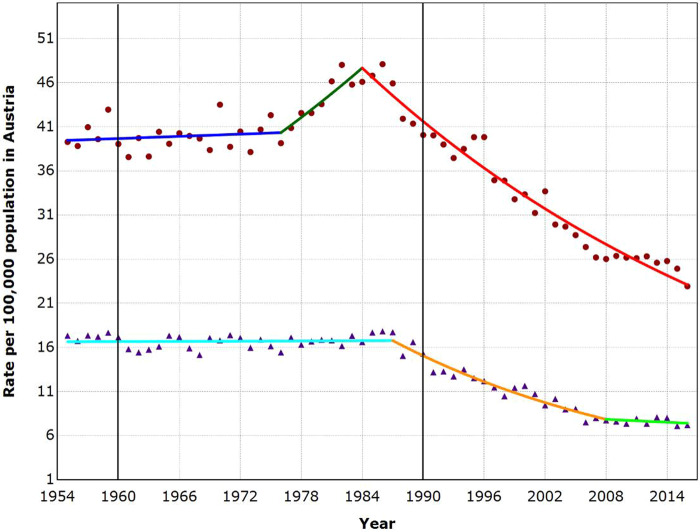
Suicide trends among males and females aged 10–89 years in Austria, 1955–2016 Dots, male suicide rate; Triangles, female suicide rate. The two bold vertical lines denote the time points when the first antidepressants were introduced (1960) and when the SSRIs were introduced and widespread prescription began (1990).

**Figure 3:**
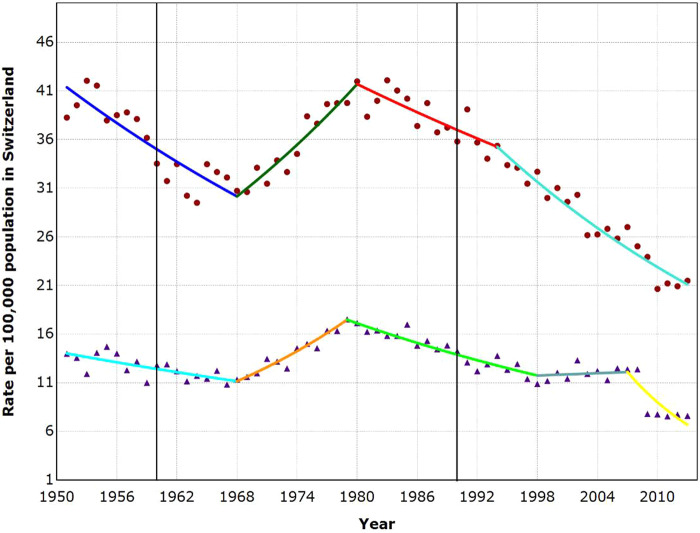
Suicide trends among males and females aged 10–89 years in Switzerland, 1951–2013 Dots, male suicide rate; Triangles, Female suicide rate. The two bold vertical lines denote the time points when the first antidepressants were introduced (1960) and when the SSRIs were introduced and widespread prescription began (1990). The last joinpoint in females is a methodological artefact due to change in reporting methods (for details, see Junker^28^).

A total of 241 708 people (175 473 males; 66 235 females) died by suicide in Italy between 1951 and 2015; 101 896 suicides (72 136 males; 29 760 females) were recorded in Austria between 1955 and 2016; and 79 876 suicides (56 611 males; 23 265 females) were recorded in Switzerland between 1951 and 2013. The average rates (per 100 000) across the entire observation period were 7.7 in Italy (males: 11.5; females: 4.1), 24.8 in Austria (males: 37.2; females: 13.8) and 23.8 in Switzerland (males: 33.7; females: 12.9). The results of the joinpoint analyses for each country, stratified by gender, are reported in table 1 and the long-term trends in suicide rates are shown in figures 1–3.

**Table 1:** APC in suicide rates in males and females according to joinpoint regression models

Country	Population	Period	APC (95% CI)
Italy	M	1951–74	−1.0 (−1.4 to −0.7)
	M	1974–82	4.2 (2.2–6.3)
	M	1982–97	0.7 (0.1–1.3)
	M	1997–2006	−2.5 (−3.9 to −1.1)
	M	2006–15	1.3 (0.1–2.5)
	F	1951–57	1.2 (−1.2 to 3.7)
	F	1957–65	−2.7 (−4.6 to −0.9)
	F	1965–85	1.8 (1.4–2.2)
	F	1985–2009	−2.1 (−2.4 to −1.9)
	F	2009–15	0.6 (−1.8 to 3.1)
Austria	M	1955–76	0.1 (−0.2 to 0.4)
	M	1976–84	2.1 (0.7–3.6)
	M	1984–2016	−2.2 (−2.4 to −2.1)
	F	1955–87	0.0 (−0.2 to 0.2)
	F	1987–2008	−3.6 (−4.0 to −3.1)
	F	2008–16	−0.7 (−2.7 to 1.3)
Switzerland	M	1951–68	−1.8 (−2.3 to −1.4)
	M	1968–80	2.7 (1.8–3.6)
	M	1980–94	−1.2 (−1.8 to −0.6)
	M	1994–2013	−2.7 (−3.0 to −2.3)
	F	1951–68	−1.3 (−2.1 to −0.6)
	F	1968–79	4.2 (2.6–5.7)
	F	1979–98	−2.1 (−2.6 to −1.5)
	F	1998–2008	0.3 (−1.7 to 2.4)
	F	2008–13	−9.4 (−12.5 to −6.2)^a^

a Joinpoint is a methodological artefact due to change in reporting methods (for details, see Junker^28^).

M, males; F, females; APC, annual percent change; CI, confidence interval.

### Trends in suicide rates—Italy

Four joinpoints were detected for each gender as depicted in figure 1. Among males, there was a significantly decreasing trend in suicide rates between 1951 and 1974 by 1.0% per year. Afterwards, the curve sharply rose by 4.2% per year until 1982 and thereafter it slightly increased by 0.7% per year until 1997. Between 1997 and 2006, a decrease of 2.5% per year was observed. Starting in 2006, a new upturn by 1.3% per year occurred until 2015. Among females, the suicide rate was stable between 1951 and 1957, then the suicide rate decreased by 2.7% per year until 1965. A new increase of 1.8% per year was recorded between 1965 and 1985. Afterwards, a decrease of 2.1% per year was reported from 1985 to 2009. Lastly, from 2009 to 2015, the rate remained stable. No decreasing trend in suicide rates began coincident with the introduction of the first antidepressants in 1960 or with the introduction and widespread prescription of SSRIs beginning 1990.

### Trends in suicide rates—Austria

As shown in figure 2, two joinpoints were found for both males and females. The shape of the trends was similar across gender. In males, the suicide rate was constant between 1955 and 1976, afterwards suicide rates increased until 1984 by 2.1% per year and then decreased by 2.2% per year until 2016. In females, the suicide rate was constant between 1955 and 1987, then the rate considerably decreased by 3.6% per year until 2008. From 2008 to 2016, the suicide rate was again constant. No decreasing trend in suicide rates began coincident with the introduction of the first antidepressants in 1960 or with the introduction and widespread prescription of SSRIs beginning 1990.

### Trends in suicide rates—Switzerland

As depicted in figure 3, joinpoint analyses indicated three joinpoints for males and four for females. In men, a decrease in suicide rates of 1.8% per year was detected between 1951 and 1968. Afterwards, the rates sharply rose by 2.7% per year until 1980. Between 1980 and 1994, the suicide rates slightly decreased by 1.2% per year and from 1994 to 2013, they fell by 2.7% per year. In women, the suicide rate fell slightly by 1.3% per year between 1951 and 1968. Afterwards there was a steep increase by 4.2% per year until 1979. From 1979 to 1998, the rates decreased again by 2.1% per year, thereafter they remained stable until 2008. From 2008 to 2013, the rates fell sharply by 9.4% per year.

However, the abrupt drop in suicide rates after 2008 is a methodological artefact due to a change in reporting methods^28^; as of 2009 physician-assisted suicides were no longer included in the Swiss suicide statistics. In fact, the stable trend starting in 1998 did not end in 2008 but continued until 2013 had the reporting methods not changed. Suicide data published by the Swiss Federal Statistical Office^28^ reveal that the female suicide rate was constant, or by tendency even slightly increasing from 2008 to 2014 when physician-assisted suicides were included in the statistic (rates per 100 000 varying between 12 and 14). As in Italy and Austria, no decreasing trend in suicide rates began coincident with the introduction of the first antidepressants in 1960 or with the introduction and widespread prescription of SSRIs beginning 1990.

## *Post-hoc* analysis

As requested during the review process, we conducted an analysis restricted to people aged 10–24 years, as various studies indicate that new-generation antidepressants increase the risk of suicidal events in youth and young adults, which also lead to a respective regulatory safety warning.^6–8^^,^^29^ The changes in long-term trends in suicide rates in people aged 10–24 years, stratified by gender, were very similar to the trends reported above for people aged 10–89 years. That is, there was no evidence that the introduction of first- or new-generation antidepressants led to sustained changes in suicide rates in people aged 10–24 years. The results of the joinpoint regression analyses for adolescents and young adults are shown in the Supplementary material.

## Discussion

Our analysis of long-term trends in suicide rates in people aged 10–89 years in Italy, Austria and Switzerland from about 1950–2015 revealed one major pattern that was largely consistent across countries and gender. Suicide rates were stable or decreasing during the 1950s and 1960s, then they rose during the 1970s, peaked in the early 1980s and thereafter they were mostly declining. However, there were a few notable gender-specific exceptions to these general trends. First, in Italian men, the suicide rates were increasing far beyond the 1980s and peaked only in 1997, thereafter they fell sharply until 2006 but increased again from 2006 to 2015. Second, in women from all three countries there was an extended period during the 2000s when suicide rates were stable. In Italian women, the suicide rates were constant from 2009 to 2015, in Austrian women, they were constant from 2008 to 2016 and in Swiss women, the suicide rates were constant between 1998 and 2013 (after correcting for a change in suicide reporting methods^28^). A *post-hoc* analysis restricted to adolescents and young adults aged 10–24 years produced comparable results.

Most importantly, the introduction of the first antidepressants around 1960 and the introduction of the SSRIs in 1990 that caused a steep increase in antidepressant prescriptions did not coincide with changes in trends of suicide rates in any country. Except for Italian men, whose suicide rates were increasing from 1974 to 1997, suicide rates were constantly declining long before antidepressant prescriptions sharply rose between 1990 and 2010.^11^ This general pattern is consistent with the findings from many other countries where negative correlations between suicide rates and antidepressant prescriptions were observed during the 1990s.^14^^,^^25^ Moreover, female suicide rates were stable over extended periods during the 2000s, despite unabated increases in antidepressant prescriptions during that period.^11^^,^^30^ These findings are consistent with long-term studies from various other countries that do not indicate that the introduction of the first antidepressants in 1960 and widespread prescription of antidepressants after the introduction of the SSRIs in 1990 had led to clear and sustained changes in suicide rates.^17–19^ Our results also agree with two systematic reviews of ecological studies, which concluded that the evidence does provide limited or no support for the hypothesis that increased antidepressant prescriptions led to reduced suicide rates in the population.^12^^,^^14^ Likewise, in adolescents and young adults aged 10–24 years there was not a consistent pattern that would indicate that the introduction of the SSRIs led to an increase in suicide rates on the population level in Italy, Austria or Switzerland.

Our findings conflict with the ecological study by Gusmao et al.^11^ who concluded that ‘These findings underline the importance of the appropriate use of antidepressants as part of routine care for people diagnosed with depression, therefore reducing the risk of suicide’ (p. 1). Although Gusmao et al.^11^ found strong negative correlations between antidepressant prescriptions and suicide rates across 29 European countries, the authors dismissed the evidence that in almost all countries the suicide rates were constantly falling long before antidepressant prescriptions exploded during the 1990s due to the introduction of the SSRIs.

Temporality is an essential premise of cause-effect relationships and refers to the necessity for a cause to precede an effect in time.^31^ Given that the decline in suicide rates beginning around 1980 in most countries (the putative effect) preceded the rise in antidepressant prescriptions after 1990 (the putative cause), logic dictates that antidepressant prescription cannot be the cause of the declining suicide rates during that period. Claiming otherwise is a logical fallacy, and this has been pointed out for many years by various researchers^12^^,^^14^^,^^25^ but seems not to have received proper recognition. To the contrary, in a rather fallacious argumentation, Gusmao et al.^11^ interpreted the declining suicide rates during the 1980s as evidence that this was an effect of first-generation antidepressant prescriptions:

‘Antidepressant utilization DDD/1000/day increase had an important effect in suicide SDR from the start, when suicide SDR started to lower in Europe. This rebuffs most criticisms and scepticism on observable antidepressant effects on suicide decrease, usually stating that suicide had already started to decrease before antidepressant utilization exploded, in the nineties, therefore denying earlier generation antidepressant effects’ (p. 11).

Gusmao et al.^11^ obviously ignored the fact that, for the few countries for which data on antidepressant use were available for the 1980s (only 5 of the 29 countries studied), the prescription rates were largely stable (Denmark and Czech Republic) or at best marginally increasing at low levels (Sweden, Norway and Finland). During the 1990s, after the introduction of the SSRIs, antidepressant use abruptly increased many times over without a discernible change in long-term trends of suicide in virtually all 29 countries (see their Figures 1–4). Moreover, since Gusmao et al.^11^ examined suicide rates only as of 1980, and not ‘from the start’ of the antidepressant treatment era, their analysis did not show that the suicide rates constantly increased during the 1970s in many European countries, as demonstrated by our data for Italy, Austria and Switzerland, but also in many other countries, e.g. Norway, Denmark and Finland.^25^ This general pattern invalidates the interpretation by Gusmao et al.^11^ that the first-generation antidepressants introduced in 1960 and the SSRIs introduced in 1990 had led to declining suicide rates. If first-generation antidepressants were responsible for the declining suicide rates during the 1980s, then, by the authors’ own logic, they would also be responsible for the increasing suicide rates during the 1970s.

Another instructive illustration comes from the USA, where declining suicide rates between 1985 and 1999 were correlated with increasing rates of antidepressant prescriptions.^10^ But while antidepressant prescriptions continued to rise during the 2000s and 2010s,^32^^,^^33^ there was a sudden trend change in suicide rates in 2000, after which the suicide rates were constantly rising in almost all age groups.^34^^,^^35^ This indicates that the negative correlation between suicide rates and antidepressant prescriptions observed between 1985 and 1999^10^ was spurious, unless the authors accept that since 2000 the ever-increasing use of antidepressants has caused a constant surge of suicides in the USA.

Therefore, we want to emphasize again how important it is to examine long-term suicide trends and to focus on the time points when antidepressants were introduced in clinical practice, instead of computing correlations between the two variables over a short timeframe. Relating suicide rates to antidepressant prescriptions over a selectively chosen short time-period can easily produce spurious correlations that lead authors to erroneous conclusions.^22^^,^^36^^,^^37^ We also want to stress that ecological studies have limited validity for assessing the effectiveness and safety of antidepressants on an individual level. The most accurate method to evaluate the benefit-risk profile of antidepressants is the clinical trial, followed by well-controlled prospective cohort studies. The results of ecological studies may help to elucidate whether antidepressants have discernible public health effects, but they must be interpreted with caution. They can neither prove nor disconfirm that antidepressants alter the suicide risk, a serious limitation that was not sufficiently acknowledged by various authors.^2^^,^^22^^,^^36^

This study has several limitations. First, due to its ecological design, the data capture only suicides at the population level and are restricted to Italy, Austria and Switzerland. The results do not necessarily generalize to other countries, although in the discussion, we emphasize similarities to various other European countries, e.g. Norway, Denmark and Finland. Second, which deaths are recorded, classified and reported as suicides may differ between countries and different time periods within countries. For instance, there is a strong correlation between autopsy rates and official suicide rates, both between and within countries, suggesting that a reduction in autopsy rates may result in misclassifications of causes of death.^38^ Third, the time points we set for the introduction of the first antidepressants in clinical practice (1960) and the sharp increase of prescriptions following the introduction of the SSRIs (1990) may slightly vary between countries. For instance, in Italy the increase of antidepressant prescriptions was rather modest during the early 1990s, but much steeper during the early 2000s.^11^^,^^24^ However, this does not alter the interpretation of our results. The nearest joinpoints following the introduction and widespread prescription of SSRIs that mark the beginning of a decreasing trend in suicide rates were 1994 in Swiss males and 1997 in Italian males (fluoxetine, the SSRI marking a new treatment era, was introduced 1991 in Switzerland and 1989 in Italy). Moreover, no changes in trends following the introduction of the SSRIs were found in Italian and Swiss females, in whom the rate of antidepressant use is about two times higher than in males.^39^^,^^40^

In conclusion, this ecological study of long-term suicide trends in Italy, Austria and Switzerland did not find evidence that the introduction of the first antidepressants in clinical practice around 1960 and the introduction of the SSRIs in 1990 that led to steep increases in prescription rates coincided with a sustained trend change in suicide rates. Except for Italian males, suicide rates were constantly declining long before antidepressant prescription rates multiplied during the 1990s, thus violating the basic premise of temporality in cause–effect relationships.^31^ These findings indicate that at the population level, the evidence does not support the popular claim that the increased use of antidepressants has contributed to a significant decline in suicide rates.^2^ Ecological studies cannot indicate cause–effect relationship and may have serious flaws,^2^^,^^22^^,^^36^ but if well conducted and cautiously interpreted they may provide valuable insights for public health. This was not always the case with ecological studies on the association between antidepressant use and suicide rates, which often focussed on selected short-term periods while neglecting long-term trends.^2^^,^^14^^,^^22^^,^^35^

## Supplementary data

Supplementary data are available at *EURPUB* online.

## Funding

No funding was received for this study.

*Conflicts of interest*: None declared.

**Key points**

- Ecological analyses restricted to short-term trends of suicide rates are often misleading.
- Long-term trends of suicide rates in the population may help to elucidate whether the introduction of antidepressants had discernible public health effects.
- Trends in suicide rates from about 1950 to 2015 in Italy, Austria and Switzerland are broadly comparable.
- With a few gender-specific exceptions, the suicide rates were stable or declining during the 1950s and 1960s, increased during the 1970s, peaked in the early 1980s and declined thereafter.
- No change in suicide trends occurred around 1960, when the first antidepressants were introduced in clinical practice or after 1990, when antidepressant prescriptions steeply rose with the introduction of the selective serotonin reuptake inhibitors.

## Supplementary Material

ckaa204_Supplementary_Data
