# Supplementary material for: Did the introduction and increased prescribing of antidepressants lead to changes in long-term trends of suicide rates?
Source: Eur J Public Health. 2020 Nov 25;31(2):291–7. doi: 10.1093/eurpub/ckaa204 (PMC8071590; doi:10.1093/eurpub/ckaa204)
Supplement: ckaa204_Supplementary_Data [file ckaa204_supplementary_data.pdf]

Supplementary material

Suicide in adolescents and young adults aged 10-24 years

A total of 19,105 young people (13,577 males; 5,528 females) died by suicide in Italy between 1951 and 2015; 9,774 suicides (7,728 males; 2,046 females) were recorded in Austria between 1955 and 2016; and 8,767 suicides (6,828 males; 1,939 females) were recorded in Switzerland between 1951 and 2013. The average rates (per 100,000) across the entire observation period were 2.6 in Italy (males: 3.6; females: 1.5), 9.9 in Austria (males: 15.3; females: 4.2), and 10.5 in Switzerland (males: 16.1; females: 4.7). The results of the joinpoint analyses for each country, stratified by gender, are reported in Table 1S and the long-term trends in suicide rates are shown in Figures 4S to 6S.

Table 1S. Annual percentage change (APC) in suicide rates in young males and females aged 10-24 years based on joinpoint regression models.

Country	Population	Period	APC (95% CI)
Italy	M	1951-1965	-3.0 (-4.1 to -2.0)
	M	1965-1989	2.1 (1.5 to 2.6)
	M	1989-1996	6.8 (3.6 to 10.2)
	M	1996-2006	-5.6 (-7.4 to -3.8)
	M	2006-2015	0.8 (-1.5 to 3.1)
	F	1951-1987	-2.9 (-3.3 to -2.5)
	F	1987-1990	11.5 (-26.3 to 68.7)
	F	1990-2015	-1.4 (-2.3 to -0.5)
Austria	M	1955-1975	0.6 (-0.1 to 1.4)
	M	1975-1978	12.8 (-10.9 to 42.8)
	M	1978-1985	1.0 (-2.4 to 4.6)
	M	1985-2016	-3.2 (-3.6 to -2.8)
	F	1955-1967	-4.6 (-7.9 to -1.2)
	F	1967-1983	3.1 (0.7 to 5.7)
	F	1983-2016	-3.0 (-3.9 to -2.2)
Switzerland	M	1951-1968	-1.1 (-2.0 to -0.1)
	M	1968-1982	4.4 (3.2 to 5.7)
	M	1982-2013	-3.5 (-3.8 to -3.1)
	F	1951-1970	-1.9 (-3.2 to -0.6)
	F	1970-1979	8.7 (4.4 to 13.1)
	F	1979-1999	-4.0 (-5.1 to -2.9)
	F	1999-2007	3.4 (-3.0 to 10.2)
	F	2007-2013	-13.2 (-20.5 to -5.3)

M: Males; F: Females; APC: annual percent change; CI: confidence interval.

Trends in suicide rates among young people – Italy

Joinpoint regression analysis detected four and two joinpoints for male and female gender, respectively (Figure 1S). Among males, there was a significantly decreasing trend in suicide rates between 1951 and 1965 by 3.0% per year. Afterwards, suicide rate increased by 2.1% per year until 1989 and sharply rose by 6.8% per year between 1989 and 1996. Between 1996 and 2006 a decrease of 5.6% per year was observed. Starting in 2006, a modest upturn by 0.8% per year occurred until 2015. Among females, the suicide rate decreased by 2.9% per year between 1951 and 1987, then the suicide rate showed an increase by 11.5% per year until 1990. Lastly, a slight decrease of 1.4% per year was recorded between 1990 and 2015.

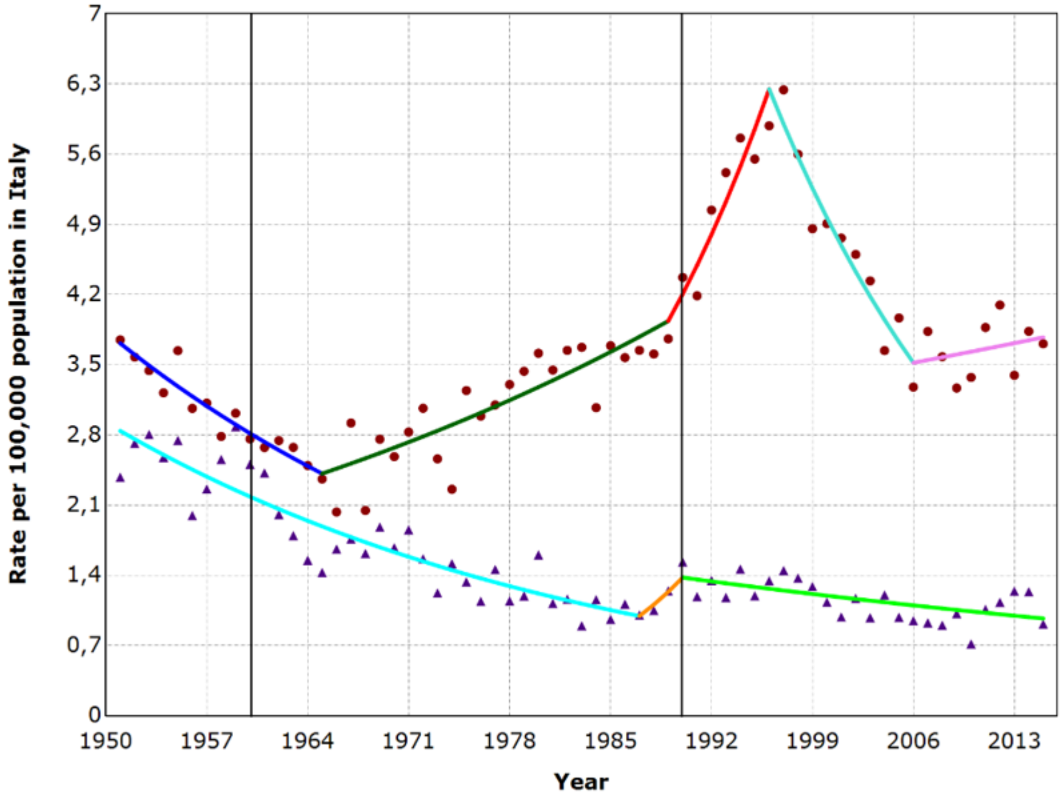

Figure 1S. Suicide trends among males (dots) and females (triangles) aged 10-24 years in Italy.

Trends in suicide rates among young people – Austria

As shown in Figure 2S, three and two joinpoints were found for males and females, respectively. In males, the suicide rate was slightly increasing by 0.6% per year between 1955 and 1975, afterwards suicide rates increased by 12.8% per year until 1978 and by 1.0% per year until 1985. Finally, suicide rate decreased by

3.2% per year between 1985 and 2016. In females the suicide rate significantly decreased by 4.6% per year between 1955 and 1967, then the rate significantly increased by 3.1% per year until 1983. From 1983 to 2016 the suicide rate decreased again by 3.0% per year.

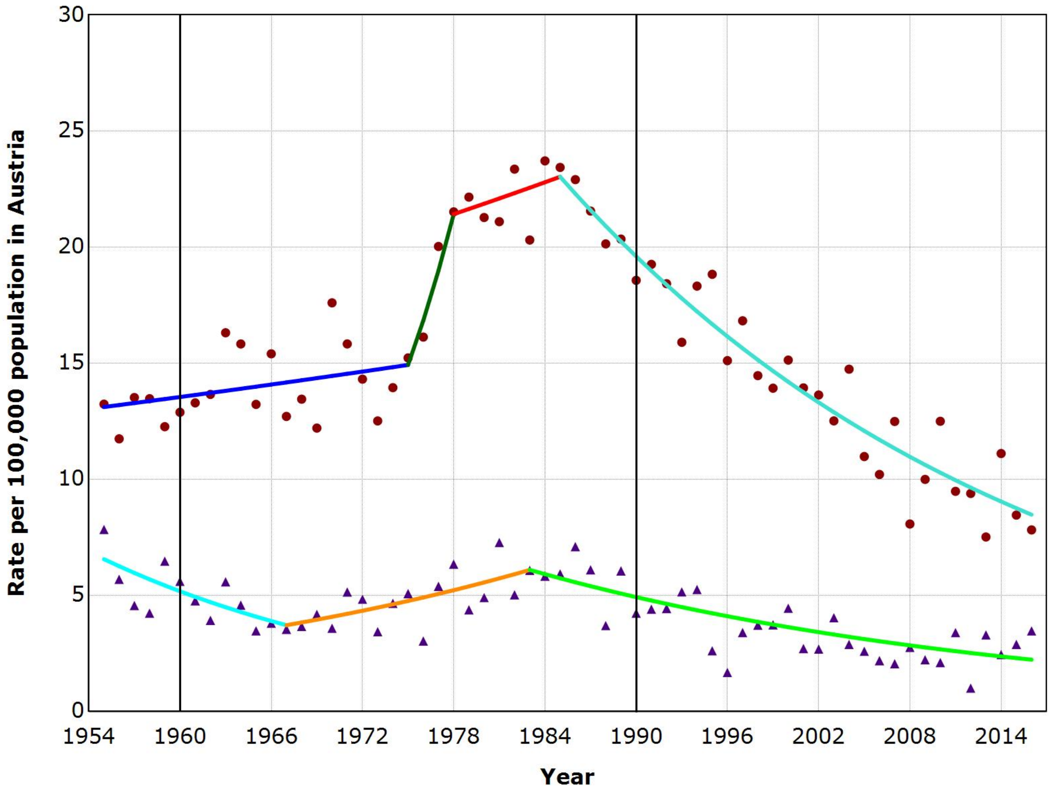

Figure 2S. Suicide trends among males (dots) and females (triangles) aged 10-24 years in Austria.

Trends in suicide rates among young people – Switzerland

As depicted in Figure 3S, joinpoint analyses indicated two joinpoints for males and four for females. In males, a small decrease in suicide rates of 1.1% per year was detected between 1951 and 1968. Afterwards, the rates sharply rose by 4.4% per year until 1982. Between 1982 and 2013 the suicide rate decreased by 3.5% per year. In female gender, the suicide rate fell by 1.9% per year between 1951 and 1970. Afterwards there was a steep increase by 8.7% per year until 1979. From 1979 to 1999 the rate decreased again by 4.0% per year, thereafter it increased by 3.4% per year until 2007. From 2007 to 2013 the rate fell sharply by 13.2% per year.

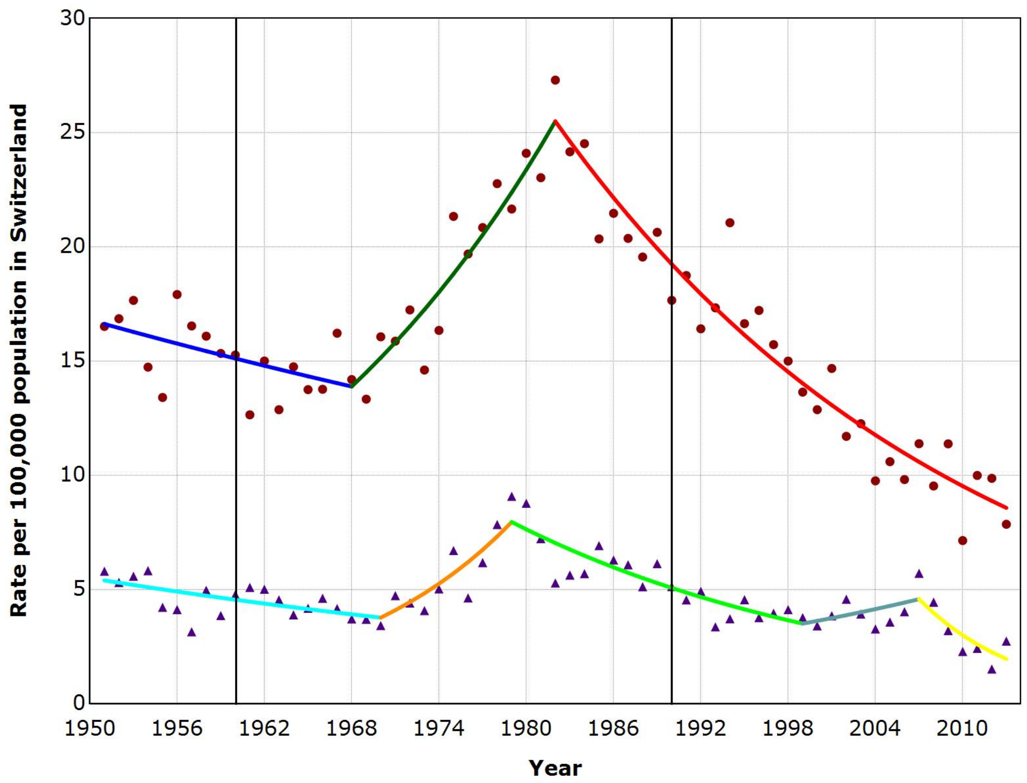

Figure 3S. Suicide trends among males (dots) and females (triangles) aged 10-24 years in Switzerland.
